# RNA Modification in Metabolism

**DOI:** 10.1002/mco2.70135

**Published:** 2025-03-10

**Authors:** Yadi Liu, Zhongyan Sun, Dingkun Gui, Yonghua Zhao, Youhua Xu

**Affiliations:** ^1^ Faculty of Chinese Medicine, State Key Laboratory of Quality Research in Chinese Medicines Macau University of Science and Technology Taipa Macao SAR PR China; ^2^ Department of Nephrology Shanghai Jiao Tong University Affiliated Sixth People's Hospital Shanghai PR China; ^3^ Institute of Chinese Medical Sciences, State Key Laboratory of Quality Research in Chinese Medicine University of Macau Taipa Macao SAR PR China; ^4^ Macau University of Science and Technology Zhuhai MUST Science and Technology Research Institute, Hengqin Zhuhai PR China

**Keywords:** epigenomics, metabolic diseases, N^6^‐methyladenosine, RNA modifications

## Abstract

Epigenetic regulation in disease development has been witnessed within this decade. RNA methylation is the predominant form of epigenetic regulation, and the most prevalent modification in RNA is N6‐methyladenosine (m^6^A). Recently, RNA modification has emerged as a potential target for disease treatment. RNA modification is a posttranscriptional gene expression regulation that is involved in both physiological and pathological processes. Evidence suggests that m^6^A methylation significantly affects RNA metabolism, and its abnormal changes have been observed in a variety of diseases. Metabolic diseases are a series of diseases caused by abnormal metabolic processes of the body, the common metabolic diseases include diabetes mellitus, obesity, and nonalcoholic fatty liver disease, etc.; although the pathogenesis of these diseases differs from each other to the current understanding, most recent studies suggested pivotal role m^6^A in modulating these metabolic diseases, and m^6^A‐based drug development has been on the agenda. This paper reviewed recent understanding of RNA modification in metabolic diseases, hoping to provide systematic information for those in this area.

## Graphical abstract

Role of m6A methylation in human metabolic diseases. A series of methylases are involved in the development of metabolic diseases; among them, FTO was found to play a pivotal role in promoting disease, YTHDC2 was believed to suppress disease development, while the role of METTL3 and METTL14 in metabolic disease still needs to be further investigated.

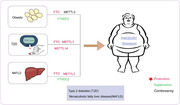

## 1 Introduction

With the acceleration of globalization, there is a clear trend toward high‐sugar and high‐fat diets [1]. This emerging trend has unfortunately precipitated a gradual and concerning increase in the prevalence of metabolic diseases [2], with conditions such as type 2 diabetes (T2D), obesity, and nonalcoholic fatty liver disease (NAFLD) becoming more commonplace [3]. These metabolic disorders not only severely impact an individual's quality of life but also impose a substantial burden on healthcare systems worldwide [4]. Furthermore, these metabolic diseases are often interrelated, contributing to the development of metabolic syndrome, which further complicates both the disease process and treatment strategies [5, 6, 7]. Currently, the treatment of metabolic diseases primarily involves lifestyle interventions such as dietary control, physical exercise, and pharmacotherapy; however, these methods have certain limitations and do not effectively cure the underlying condition [8, 9, 10]. Therefore, it is of great clinical significance and social value to deeply investigate the pathogenesis of metabolic diseases and identify new therapeutic targets and intervention strategies [11, 12]. In recent years, the scientific community has been actively exploring the underlying mechanisms of these diseases [9, 13].

Recent studies have intriguingly proposed a potential link between RNA modifications and the development of metabolic diseases [2, 14]. RNA modifications encompass a broad spectrum, with m^6^A being one of the most significant forms [15, 16, 17]. In metabolic disorders, aberrant RNA modifications can disrupt gene expression [18]. Taking m^6^A as an example, alterations in its modification levels can impact mRNA stability and translation efficiency [19, 20]. For instance, in diabetes, abnormal m^6^A modifications on the mRNA of genes involved in key metabolic pathways may lead to changes in the expression of proteins related to insulin secretion, subsequently affecting blood glucose metabolism [21, 22, 23]. These RNA modifications are anticipated to serve as potential biomarkers for early diagnosis of metabolic diseases and may also be developed as therapeutic targets, offering new avenues for intervention in these conditions [24, 25].

However, research into the relationship between metabolic diseases and RNA modifications remains in its nascent stages. While significant findings have been achieved, numerous aspects require further investigation [26, 27]. For instance, the precise mechanisms by which various RNA modifications influence metabolic diseases are not yet fully elucidated. Questions remain regarding potential interactions between different RNA modification types and how these interactions might affect disease onset and progression. Additionally, there is a need to explore how RNA modifications can be harnessed as therapeutic targets to develop novel treatment strategies for metabolic diseases [28, 29]. Addressing these challenges will pave the way for innovative approaches to prevent and treat metabolic disorders [30]. It must be recognized that the current comprehension of RNA modifications’ role in the development of metabolic diseases is still fragmented [31]. A more comprehensive and systematic understanding is imperative to fully leverage the potential of RNA modification research in combating metabolic diseases.

In fact, a significant number of noncoding RNAs, such as rRNAs and tRNAs, undergo extensive modifications [32]. m^7^G caps [33], poly(A) tails [34], and N6‐methyladenosine (m^6^A) are the most frequent RNA modifications in addition to conventional terminal modifications [35, 36]. It is estimated that over 300 noncoding RNAs and 7000 coding RNAs contain m^6^A [37]. There are various types of RNA modifications [38, 39], and some common modifications include m^6^A, 5‐methylcytosine (m^5^C), N1‐methyladenosine (m^1^A), and pseudouracil (Ψ) [40, 41]. m^6^A, the most common internal modification in eukaryotic mRNA, is the addition of a methyl group to the adenine nitrogen atom at position 6 of the mRNA [42, 43]; moreover, m^6^A is considered a dynamic and reversible modification that plays an important role in regulating gene expression, RNA splicing, RNA stability, and RNA editing [44, 45]. Its regulators often involve writer, eraser, and reader proteins [46, 47, 48]. Recent studies have illuminated the potential connection between aberrant m^6^A methylation and various biological processes [49]. Increasing evidence indicates that abnormal m^6^A methylation can impair normal cellular functions and may contribute to the onset of metabolic diseases [50, 51, 52]. Consequently, a thorough investigation into the mechanisms and roles of m^6^A modification is highly promising [53], potentially offering new perspectives and strategies for managing these complex metabolic disorders, thereby paving the way for novel therapeutic approaches in the future.

This review emphasizes the pivotal role of RNA modifications in metabolic processes. To facilitate a comprehensive understanding, the paper initially provides an overview of various forms of RNA modifications and subsequently summarizes those non‐m^6^A modifications associated with metabolic diseases. Then, it overviewed the most frequent RNA modification “m^6^A” from the perspective of its discovery and detection method progression, and then discussed the mechanism of m^6^A modification and the role of this modification in the development of metabolic diseases. Simultaneously, it elucidated the connection between aberrant RNA modifications and metabolic disorders such as diabetes, obesity, and metabolic syndrome. Furthermore, it outlined novel disease diagnostic and therapeutic strategies grounded in RNA modification research, thereby offering a comprehensive reference for ongoing investigations in the metabolic field. It is hoped that this paper will introduce fresh perspectives for both the study and treatment of metabolic diseases.

## 2 RNA Modification and Metabolic Diseases

### 2.1 RNA Modification and RNA Methylation

Epigenetic regulation is a crucial and mysterious research field in the field of life science, which covers a variety of exquisite and complex mechanisms, mainly including histone modification, DNA modification, and noncoding RNA modification [54, 55]. One such mechanism is posttranscriptional modification of RNA, which involves altering the chemical composition of the RNA molecule [56, 57]. In recent years, with the rapid development of scientific research technology, this form of regulation has gradually emerged and is widely regarded by the scientific community as an important and unique means to control gene expression [58]; in fact, over 170 different types of RNA chemical modifications having been reported [38, 59, 60]. These intricate and diverse RNA modifications are pivotal in the nuanced landscape of RNA metabolism [61]. They can influence various facets, including the stability of mRNA transcripts, the nuclear export dynamics of RNA molecules, translation efficiency, and the precision of decoding processes [30, 62]. Through meticulous regulation of these pathways, RNA modifications significantly shape the ultimate outcome of gene expression [63]. Investigating RNA modifications offers a window into comprehending the intricacies of gene expression regulation and how it modulates in both physiological and pathological contexts [25].

The current body of research consistently shows the dominance of RNA methylation across a wide range of RNA modification types [64, 65, 66]. The underlying principle of RNA methylation is similar to that of DNA methylation, where a methyl group is covalently attached to different N or C positions of bases [67]. Given the diverse range of RNA molecules, including tRNA, mRNA, and rRNA, the functions of methylated RNA are highly versatile [68, 69]. Given the vast array of RNA molecules, the functions of methylated RNA exhibit considerable diversity. In metabolic diseases such as diabetes and obesity, irregular alterations in RNA methylation patterns are frequently observed, potentially leading to the misregulated expression of genes involved in metabolism [70]. These disruptions subsequently trigger a cascade of metabolic disorders, ultimately facilitating the onset and progression of these diseases.

### 2.2 Types of RNA Methylation Modifications

**FIGURE 1:**
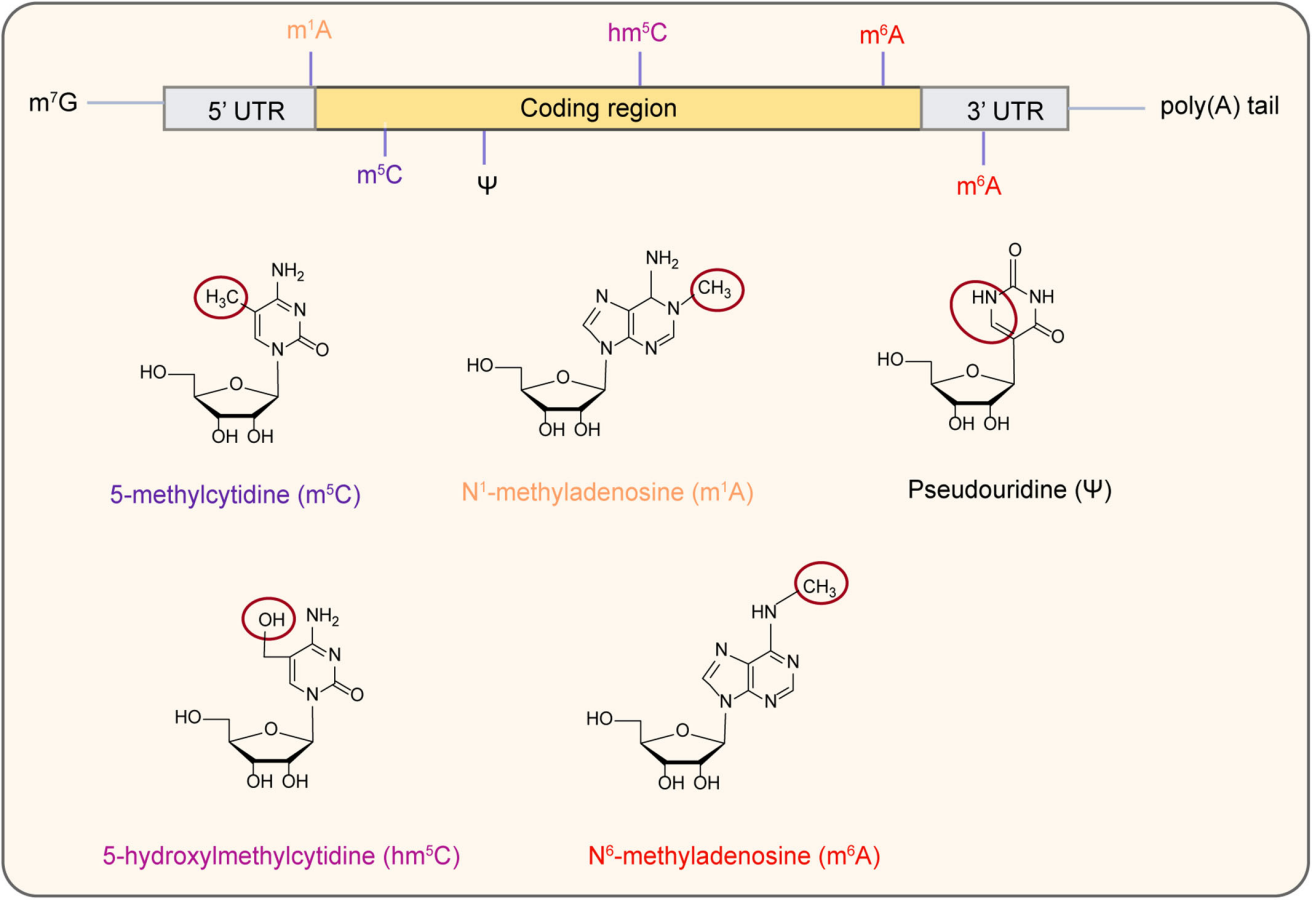
Common chemical modifications in mRNA transcripts. The mRNAs with poly(A) tails and m^7^G‐caps are indicated with blue lines. m^5^C, m^6^A, m^1^A, hm^5^C, and Ψ modifications are shown schematically (top) with their chemical structures (bottom).

Within mRNA, several nucleosides with base modifications have been identified (Figure 1), including the modification of 5‐methylcytosine (m^5^C), N1‐methyladenosine (m^1^A), pseudouridine (Ψ), 5‐hydroxymethylcytosine (hm^5^C), and N6‐methyladenosine (m^6^A), N7‐methylguanosine (m^7^G), N4‐acetylcytosine (ac^4^C), and other modifications. m^5^C is a methylation modification on the fifth carbon atom of cytosine in RNA molecules and m^5^C in RNA can be oxidized to hm^5^C by Tet family enzymes, m^1^A is a methylation modification on the first nitrogen atom of adenosine in RNA molecules, and Ψ is an isomer of uridine in RNA molecules [71, 72].

RNA methylation modifications come in various types, each fine‐tuning RNA function through unique mechanisms and subsequently influencing diverse physiological processes and cellular activities [73, 74]. A deeper understanding of these RNA methylation modifications will not only unveil the complexities of life processes but also offer new targets and perspectives for disease diagnosis, treatment, and drug development. As technology advances, it is anticipated that more novel discoveries in RNA methylation modification will pave the way for significant breakthroughs and advancements in the field of life sciences.

### 2.3 Common RNA Modifications (Non‐m^6^AModification) and Obesity

Obesity has emerged as a significant global public health challenge, impacting not only individual physical health but also closely associating with the onset and progression of numerous chronic conditions [75, 76]. In recent years, advancements in molecular biology technology have facilitated deeper investigations into the mechanisms underlying obesity. Notably, RNA modifications have been implicated in the development of metabolic disorders, including obesity [28, 77]. Among these modifications, m^5^C (N^6^‐methyladenosine) stands out as a prevalent form, potentially influencing obesity through its regulation of genes involved in lipid metabolism. For instance, Liu et al. [78] demonstrated that m^5^C modification of mRNA can control adipogenesis by enhancing the export and translation of CDKN1A mRNA, thereby regulating cell cycle progression and highlighting the pivotal role of m5C in adipogenesis, thus offering a potential therapeutic target for obesity prevention. Further studies have underscored the critical function of m^5^C in modulating both adipogenesis and myogenesis, suggesting it as a promising therapeutic avenue for addressing obesity, skeletal muscle dysfunction, and metabolic disorders [79]. In the context of obesity research, alterations in these modifications may disrupt energy homeostasis, ultimately contributing to weight gain and obesity [58, 74].

Certain RNA modifications, which have been the subject of relatively limited research, may also contribute to obesity development. Leveraging our deepening comprehension of RNA modifications could facilitate the development of targeted or therapeutic agents aimed at correcting aberrant RNA modifications, thereby restoring metabolic equilibrium and mitigating obesity—a development with profound implications for novel therapeutic strategies against obesity [80]. Nonetheless, the exploration of the correlation between prevalent RNA modifications and obesity remains nascent, with numerous challenges yet to be addressed. For instance: How can we effectively target RNA modifications for clinical intervention? Do RNA modifications exhibit distinct roles across different tissues and organs [81, 82]? It is anticipated that as research progresses, our understanding of RNA modifications in relation to obesity will become more comprehensive and nuanced, offering fresh perspectives and methodologies for the effective prevention and treatment of obesity and significantly contributing to the enhancement of human health.

### 2.4 Common RNA Modifications (Non‐m^6^A Modification) and T2D

Type 2 diabetes mellitus (T2D) has emerged as a pervasive global health issue, posing a severe threat to human well‐being and necessitating comprehensive strategies for prevention and management. Its etiology is intricate, encompassing insulin resistance, impaired function of pancreatic β‐cells, and disturbances across multiple metabolic pathways [83, 84]. As epigenetic research advances, mounting evidence underscores the pivotal role of RNA modifications—a critical epigenetic regulatory mechanism—in the initiation and progression of T2D [85, 86, 87]. Notably, beyond m^6^A modification, other forms such as m^5^C modifications are indispensable in RNA metabolism, functionality, and cellular physiological processes, and they bear a close association with the pathogenesis of T2D. For instance, Song et al. [88] have elucidated that m^5^C‐related genes exhibit markedly differential expression in T2D, thereby identifying potential biomarkers and therapeutic targets for this condition.

A comprehensive comprehension of the interplay between these RNA modifications and T2D is anticipated to unveil novel targets and strategies for the diagnosis, treatment, and prevention of this condition [86]. It is anticipated that as research into the relationship between RNA modifications and T2D progresses, it will yield new breakthroughs, potentially transforming our understanding of the disease's mechanisms. These advancements may offer renewed hope for the prevention, diagnosis, and therapy of T2D, paving the way for more effective treatments and improved patient outcomes.

### 2.5 Common RNA Modifications (Non‐m^6^A Modification) and NAFLD

NAFLD encompasses a spectrum of hepatic conditions, extending from simple hepatic steatosis to nonalcoholic steatohepatitis (NASH), and potentially progressing to cirrhosis and hepatocellular carcinoma, posing significant health risks if left untreated [89]. As the prevalence of global obesity rises, so does the incidence of NAFLD, positioning it as a significant public health challenge [90]. While there is a growing understanding of the pathogenesis of NAFLD, including insulin resistance, lipid metabolism disorders, and oxidative stress, numerous aspects remain unexplored and necessitate further investigation [91].

RNA modification serves as a pivotal epigenetic regulatory mechanism, significantly influencing gene expression regulation [92]. Beyond the extensively studied m^6^A modification, an array of other RNA modifications also play crucial roles in cellular physiological and pathological processes. Notably, the significance of m^1^A modification has increasingly come to the forefront. A study demonstrated for the first time that m^1^A methylation modification plays a crucial role in the occurrence and development of metabolic dysfunction‐associated fatty liver disease, and they suggested that it may lead to reprogramming of the immune microenvironment and trigger metabolic inflammation in the liver [93].

Growing evidence indicates a strong correlation between these RNA modifications and the onset and progression of NAFLD. Delving deeper into this relationship could illuminate the pathogenesis of NAFLD and open new avenues for its therapeutic intervention. However, future research is essential to fully unravel the mechanisms by which different RNA modifications contribute to NAFLD, particularly their interactions and specific roles in various liver cell types. Additionally, there is an urgent need to develop more precise techniques for detecting RNA modifications and effective intervention strategies. It is anticipated that as research into the interplay between RNA modifications and NAFLD progresses, it will yield novel insights and breakthroughs in the prevention, diagnosis, and treatment of NAFLD.

### 2.6 Potential Application of RNA Modifications (Non‐m^6^A Modification) in Metabolic Diseases

Given that RNA modifications are uniquely altered in metabolic disorders, they exhibit significant potential as diagnostic biomarkers [70, 94]. The extent of mRNA modification in certain metabolic‐related genes can serve as promising indicators for metabolic diseases [95]. For instance, the m^5^C modification levels of particular genes in diabetic patients diverge from those found in healthy controls, and by monitoring these modification levels, early detection of diabetes may become feasible [88]. In cases of NAFLD or obesity, RNA modifications could be leveraged for both disease diagnosis and condition assessment. Analyzing the expression of enzymes involved in RNA modification within tissues, along with the overall RNA modification profile, allows for a more precise determination of the disease's type and severity [96, 97].

Consequently, beyond m^6^A modification, a variety of other RNA modifications are pivotal in the onset and progression of metabolic diseases [98]. They contribute to the regulation of metabolism‐associated gene expression via diverse mechanisms and influence cellular metabolic functions. These RNA modifications have demonstrated considerable promise for diagnostics and therapeutic interventions in metabolic disorders, heralding them as prospective biomarkers and therapeutic targets. Consequently, the development of specific modulators targeting RNA modifications represents a promising therapeutic avenue for diseases like NAFLD. Nonetheless, when formulating such drugs, their selectivity and safety must be meticulously assessed to prevent detrimental effects on normal cellular RNA modifications. Presently, research into these RNA modifications in the context of metabolic diseases is nascent and fraught with numerous challenges and unresolved issues. Regarding mechanistic understanding, while some advancements have been made, many details remain elusive, necessitating further in‐depth investigations to elucidate the interplay between RNA modifications and metabolic disease‐related signaling pathways. The complexity of RNA biology adds an additional layer of difficulty, as it involves intricate regulatory networks that are not fully understood. Moreover, the dynamic nature of RNA modifications under varying physiological conditions poses significant hurdles for researchers. Despite these obstacles, ongoing studies are gradually unveiling new insights, paving the way for potential therapeutic interventions targeting RNA modifications to combat metabolic disorders effectively.

Among types of modifications, m^6^A is the most frequent RNA modification [99, 100]. Given the ubiquity of m^6^A in RNA modifications and its pivotal role in the emergence and progression of metabolic diseases, an in‐depth exploration of m^6^A and its association with metabolic disorders holds significant scientific importance and clinical relevance. Therefore, this paper will further concentrate on m^6^A and metabolic diseases, aiming to deliver a thorough and exhaustive overview. This endeavor is intended to furnish a more nuanced theoretical foundation and a wealth of research ideas for scholars and practitioners navigating the realms of related fields. By delving deeper into the mechanisms by which m^6^A modifications influence metabolic processes, this review aims to shed light on previously overlooked connections between RNA biology and metabolic disorders. The comprehensive analysis will encompass both established findings and emerging trends, providing readers with actionable insights that could spur new avenues of investigation and potentially lead to innovative therapeutic strategies.

## 3 Overview of m^6^A Methylation

### 3.1 Discovery of M^6^A Modification

The m^6^A modification refers to the methylation of the nitrogen atom at the sixth position of RNA adenine [101, 102, 103], and this modification will result in the formation of methylated adenosine and further modulate gene expression [104]. Recent studies have indicated that m^6^A modification is the most abundant form of RNA methylation [105, 106, 107]. This underscores its critical role in cellular physiological activities and its extensive involvement in diverse biological processes [108, 109].

The m^6^A modification was first identified in the 1970s [110, 111], but its function and mechanism remained largely unexplored until recent years. The discovery of m^6^A modification represents a significant milestone in the field of life sciences, paving the way for novel insights into the mechanisms underlying genetic information transmission and regulation. In 2011, the discovery of the first genuine m^6^A demethylase associated with fat mass and obesity (FTO) sparked renewed interest in mRNA methylation [112]. This breakthrough revealed that m^6^A is a reversible modification and has significant implications for health and disease development [113]. Since then, researchers have been working hard to explore the complex role of m^6^A in various biological processes, including gene expression regulation, RNA splicing, and translation [114, 115]. In fact, the study of m^6^A has opened up a new avenue for understanding the molecular mechanisms underlying human diseases and developing novel therapeutic strategies [116, 117, 118]. However, the lack of reliable analytical methods has been a major obstacle to the in‐depth study of m6A. In the early studies, scientists' research on the specific mechanism and biological function of m6A modification was greatly limited due to the inability to accurately identify and locate the m6A modification sites on RNA molecules. They can only speculate on the presence and possible role of m6A modification by some indirect methods, but these methods are often not accurate and comprehensive enough.

In 2012, the emergence of the methylated RNA immunoprecipitation sequencing (MeRIP‐Seq) method provided a strategy to explore the modification effect of m^6^A. This breakthrough allowed researchers to identify over 12,000 m^6^A loci with typical consensus signatures in transcripts of more than 7000 genes in humans, highlighting its crucial role in regulating gene expression [119]. Since then, research on m^6^A modification has made remarkable progress [120].

As research into m^6^A modification deepens, an increasing number of its mysteries are being unveiled. This advancement not only sheds light on the mechanisms of genetic information transmission and regulation but also opens up new avenues for combating human diseases, including tumors, neurological disorders, and metabolic conditions [105, 121, 122]. Furthermore, with continuous technological progress and innovation, more precise and efficient research methods are being developed, which in turn accelerate the advancement of studies in the realm of m^6^A modification, enhancing our understanding and potential applications.

### 3.2 Technical Progress for Detection of M^6^A Modification

The study of the m^6^A modification function starts with selecting an appropriate method to detect or predict m^6^A [123, 124]. However, progress in detecting and quantifying m^6^A has been slow due to technical limitations. The advancements in MS sensitivity and high‐throughput sequencing have promoted and enriched detection methods for m^6^A [125]. Currently, m^6^A detection is primarily conducted through liquid chromatography, mass spectrometry (LC‐MS), and high‐throughput sequencing [126]. Although LC‐MS/MS can detect the overall level of mRNA for m^6^A, it cannot determine the exact location of the modification [127, 128]. Therefore, high‐throughput sequencing remains the primary method for studying m^6^A modification at present [50, 129].

The MeRIP‐Seq method was first introduced in 2012 by Meyer et al. [119], who developed an immunoprecipitation‐based approach for m^6^A localization throughout the transcriptome using methylated RNA sequencing (MeRIP‐Seq). This study identified m^6^A as a prevalent mRNA modification in 7676 mammalian genes. Another technique called m^6^A‐seq was proposed by Dominissini et al. in 2012, which detected over 12,000 m^6^A sites with classic consensus characteristics in more than 7000 human gene transcripts [130]. As research into m^6^A modification deepens, it has emerged as a pivotal player in various biological processes, including gene expression regulation, RNA splicing, mRNA stability, and translation efficiency. The advent of MeRIP‐seq and m^6^A‐seq technologies has undeniably accelerated our functional studies in this field. These advanced techniques enable researchers to rapidly identify genes and biological pathways associated with m^6^A modification, significantly enhancing our understanding of its functions by providing deeper insights into the molecular mechanisms at play. MeRIP‐seq and m^6^A‐seq are two techniques that employ specific antibodies to enrich m^6^A‐modified RNA fragments and purify mRNA fragments for sequencing. As first‐generation technologies, they have been instrumental in providing guidance for the analysis of m^6^A distribution across the transcriptome and accelerating the functional study of this modification. However, no technique is perfect, and MeRIP‐seq and m6A‐seq are no exception, the resolution for these methods is low, which makes it difficult to identify single‐base changes [131]. The limitations of the first‐generation technologies such as MeRIP‐seq and m6A‐seq restrict the study of m^6^A modification, with advances in detection methods, this limitation has gradually been overcome. In 2015, Linder et al. [132] introduced a novel approach called miClip‐seq. This technique involves the use of m^6^A‐specific antibodies and UV cross‐linking to map m^6^A residues, resulting in unique signature mutations. By comparing the sequencing analysis with known genome sequences, the precise location of m^6^A residues can be determined, and single‐base resolution was realized. The advent of this technology marks a new era in the study of m^6^A modification, offering a potent tool for uncovering the intricate mechanisms behind m6A's role in gene expression regulation, disease onset, and progression. This advancement significantly propels the development of related fields.

Besides antibody‐dependent techniques, some antibody‐independent methods have also been developed. In 2019, a highly accurate and high‐throughput m^6^A identification method was developed that does not rely on antibodies. This method, known as m^6^A‐REF‐seq [133], utilizes a m^6^A‐sensitive RNA endonuclease to facilitate sequencing, and the resolution was further elevated. m^6^A‐REF‐seq, as an antibody‐independent method for m^6^A identification, brings a breakthrough for the study of m^6^A modification with its unique technical advantages. DART‐seq is another antibody‐independent method that targets and labels the entire transcriptomic RNA m^6^A sites originating from cellular metabolism using m^6^A‐label‐seq [134]. This strategy allows for single‐base resolution determination by subjecting the labeled sites to chemical treatment‐induced reverse transcription base mutations [135]. Additionally, a recently reported method known as m^6^A‐SAC‐Seq has gained attention due to its ability to cover almost all m^6^A classical motifs and directly label m^6^A. Furthermore, it can quantitatively analyze the captured m^6^A sites with single‐base resolution, making it a valuable tool in the study of m^6^A modifications [136]. In 2023, Wang et al. introduced a novel approach called GLORI (glyoxal and nitrite‐mediated deamination of unmethylated adenosine), a newly developed m^6^A detection technology which provides the ability to identify m^6^A at the single‐base level. In the previous m^6^A detection techniques, the quantitative analysis often has large errors, which makes it difficult to accurately reflect the true level of m6A modification in the sample. This technology can absolutely quantify single‐base m^6^A methylation with high specificity and sensitivity, and is an important progress in current m^6^A detection technology [137]. It not only fills the gap of traditional techniques but also opens up a new way in the field of m^6^A modification research.

Progress of m^6^A detection strategies obviously promoted the discovery of its modification mechanism as well as its role in disease development [138, 139]. The discovery of m^6^A modification is a gradual and in‐depth process, evolving from the initial chemical detection of its existence in early stages to the use of high‐throughput sequencing technology that reveals its distribution characteristics. Subsequently, the identification of related proteins and their functions in biological development and diseases have been thoroughly investigated [140, 141, 142]. Each stage has been marked by technological advancements and research breakthroughs. Currently, the detection technology for m^6^A modification has made significant progress, providing a powerful tool for an in‐depth study of its biological functions. Different detection techniques each have their own advantages and disadvantages; therefore, it is essential to choose the appropriate method based on the research purpose and sample characteristics.

Although the study of m^6^A modification offers new insights into understanding the regulation mechanisms of gene expression and provides new targets and ideas for research and treatment in related fields due to its important role in biological development and diseases [143, 144, 145], there are still many unknown areas waiting to be explored. For instance, the precise regulation of m^6^A modification to treat related metabolic diseases remains an area requiring further research. In the future, the development direction of m^6^A modification detection technology may involve improving the accuracy and resolution of detection, reducing detection costs, and expanding the application range of this technology. Additionally, the integration of multi‐omics technologies—such as combining m^6^A modification detection with transcriptomics and proteomics—will enable a more comprehensive understanding of the role of m^6^A modification in gene expression regulatory networks. This approach will also provide new targets and strategies for biomedical research and disease treatment (Table 1).

**TABLE 1:** Summary of m^6^A detection strategies.

Sequencing method	Time	Features	References
MeRIP‐seq m^6^A‐seq	2012	Antibody‐dependent. Large range but unable to quantify.	[119, 130]
miClip‐seq	2015	Single‐base precision detection of m^6^A sites. Low cross‐linking rate.	[132]
m^6^A‐REF‐seq	2019	Directly detectable. Only responds to m^6^ACA sequences.	[133]
DART‐seq	2019	Depends on cell transfection efficiency.	[134]
m^6^A‐label‐seq	2020	Single‐base resolution, but labeling yield requires optimization.	[135]
m^6^A‐SAC‐seq	2022	Directly labels m^6^A and quantifies captured m^6^A sites with single‐base resolution.	[136]
GLORI‐seq	2023	High efficiency, high sensitivity, high specificity, no preference for single base m^6^A site detection.	[137]

**TABLE 2:** The role of m^6^A modification in obesity.

m6A methylase	Main functions	References
FTO	Promotes autophagy and lipogenesis via autophagosome formation. Promotes NADP‐enhanced adipogenesis in 3T3‐L1 preadipocytes.	[216] [217]
METTL3	Suppression of obesity and systemic IR via promoting the maturation of BAT. Promotes obesity and NAFLD in mice via regulation of m^6^A‐modified DDIT4.	[219]
YTHDF2	Reduces mRNA degradation and protein expression thereby alleviating lipogenesis.	[216]

## 4 Mechanism of m^6^A modification

### 4.1 m^6^A Methylases: Writers, Erasers, and Readers

As discussed above, m^6^A is a methylation modification on RNA which has an important impact on RNA splicing, stability, localization, and translation. The formation of m^6^A requires the assistance of m^6^A methyltransferases, also known as writers [146, 147, 148]. Currently, a number of writers have been identified, including METTL3, METTL14, and WTAP, with METTL3 being the first to be discovered [149, 150, 151]. The primary function of writers is to catalyze adenylate methylation, and they often work in complex with other m^6^A methyltransferases [152, 153, 154]. In contrast, m^6^A demethylases such as FTO [155, 156, 157] and ALKBH5 [158] are responsible for the removal of methyl groups from m^6^A‐modified RNA, a process known as demethylation [159]. These enzymes are commonly referred to as erasers due to their ability to erase m^6^A marks on RNA [160].

The presence of writers and erasers indicates that m^6^A modifications are reversible, and readers play a crucial role in interpreting the signals encoded by m^6^A binding proteins [146, 161]. These readers include the YT521‐B homeodomain family proteins YTHDF1/2/3 [162] and insulin‐like growth factor 2‐mRNA binding proteins such as IGF2BP1 [163], IGF2BP2, and IGF2BP3 [164]. The information contained within m^6^A modifications is translated into functional signals that regulate gene expression and cellular processes.

**FIGURE 2:**
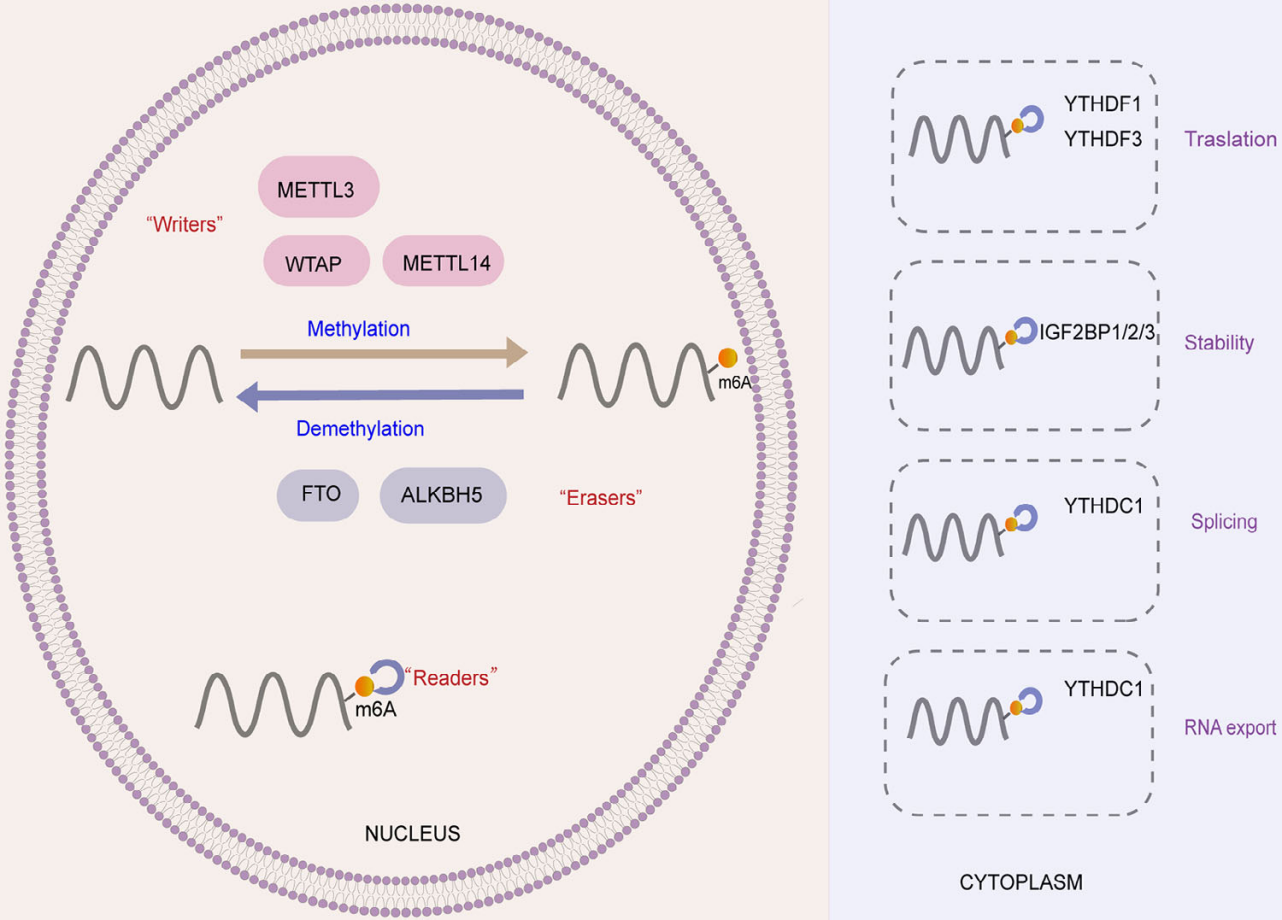
The reversible and dynamic m^6^A RNA modification and mechanisms of m^6^A modification. The diagram on the left illustrates the reversible nature of m^6^A modifications, where writers such as METTL3, METTL14, and WTAP install m^6^A marks. On the other hand, erasers like FTO and ALKBH5 have the ability to reverse this modification. The diagram on the right highlights the role of readers in determining the impact of m^6^A on mRNA processing, splicing, stability, and translation. Readers such as YTHDF1/2/3 and IGF2BP1/2/3 play a crucial role in interpreting these modifications.

The collaboration of these m^6^A methylases can potentially modify RNA expression (Figure 2), thereby modulating the development and progression of metabolic diseases.

**FIGURE 3:**
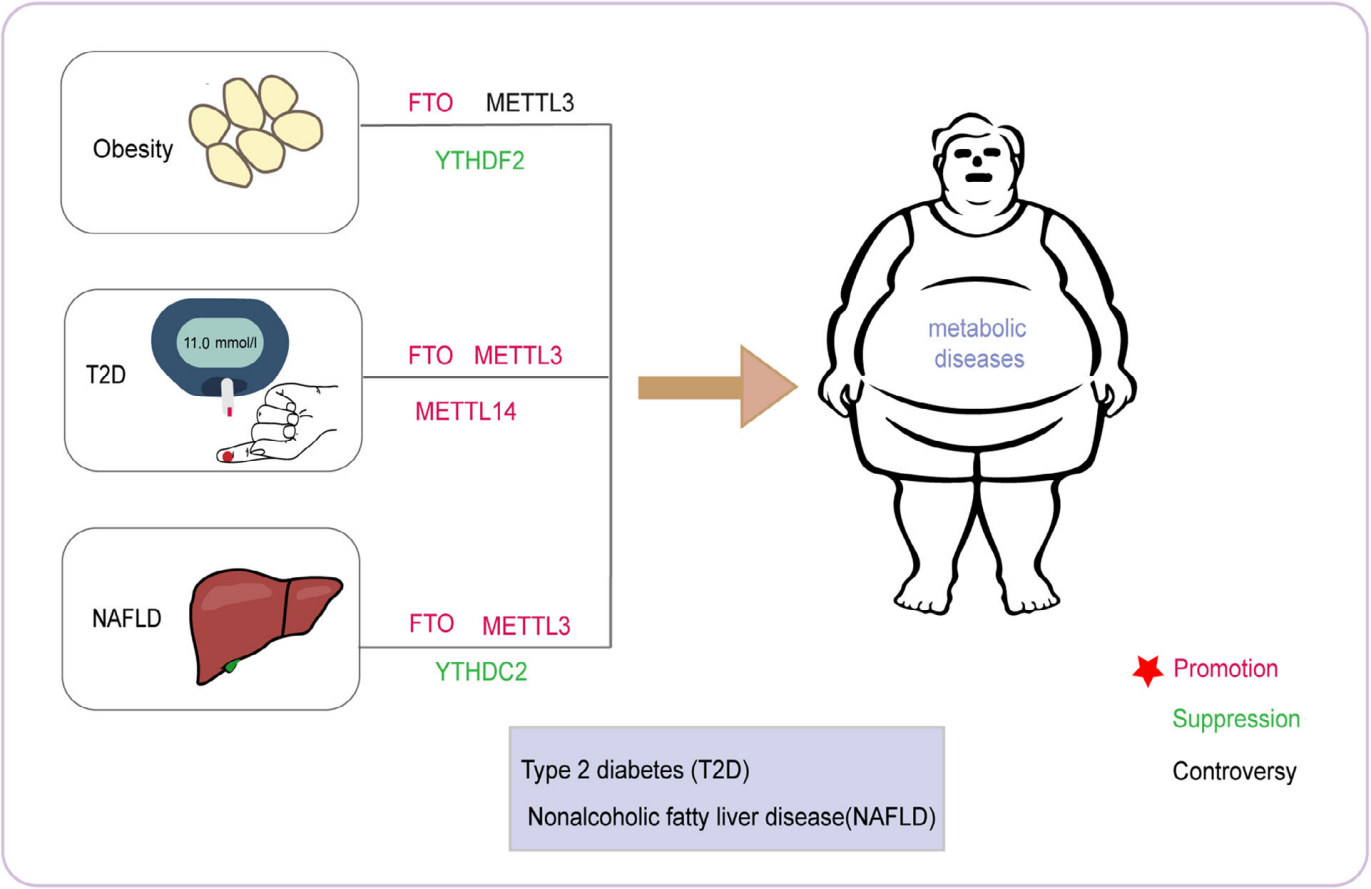
Role of m6A methylation in human metabolic diseases. Obesity is a complex disease that involves multiple genetic and environmental factors. Recent research has identified three main methylases, FTO, METTL3, and YTHDF2, that play a role in the development of obesity. FTO expression is upregulated in obese individuals and promotes the formation of obesity. In contrast, YTHDF2 expression is downregulated and acts as an inhibitor in the progression of obesity. The role of METTL3 in obesity remains controversial. T2D is another metabolic disorder that shares some common genetic factors with obesity. The main methylases involved in T2D are FTO, METTL3, and METTL14. Both METTL3 and METTL14 are upregulated and have a facilitating role in the progression of T2D. NAFLD is a liver disease that is closely linked to obesity and T2D. The main methylases involved in NAFLD are FTO, METTL3, and YTHDC2. FTO and METTL3 expressions are upregulated in NAFLD, promoting its progression. YTHDC2 expression is downregulated in NAFLD, further contributing to its pathogenesis. In conclusion, understanding the role of these methylases in the development of metabolic disorders may lead to new therapeutic targets for treating these diseases.

### 4.2 Regulation of RNA Biological Processes Involved in M^6^A Modification

m^6^A is a reversible and dynamic modification that plays a crucial role in regulating mRNA by impacting its stability, splicing, exportation, and translation [82, 165, 166] (Figure 2). During this process, a series of proteins are involved.

YTHDC1, YTHDC2, YTHDF1, YTHDF2, and YTHDF3 are members of the same family characterized by their YTH structural domains, but each serves distinct physiological functions [167, 168, 169]. Recent studies suggested that m6A modification is strictly modulated by these proteins. YTHDF1 plays a crucial role in promoting mRNA translation by binding to the translation initiation complex [170, 171, 172]. This process involves recognition of m^6^A modifications on mRNA by YTHDF1, which then actively contributes to protein synthesis [173]. By working in tandem with the translational machinery, YTHDF1‐mediated translation enhances translation efficiency, ensuring that m^6^A‐tagged dynamic transcripts produce efficient proteins. The m^6^A‐modified target RNA is exported from the nucleus to the cytoplasm, where it may first be recognized by either YTHDF3 or the YTHDF3‐YTHDF1 complex. Subsequently, YTHDF3 synergizes with YTHDF1 to promote translation and regulate m^6^A‐dependent mRNA decay via YTHDF2. In addition to its role in translation, YTHDF3 is also essential for promoting metabolism in conjunction with YTHDF1 and YTHDF2 [174].

Pre‐mRNA splicing is a crucial gene regulatory process that plays a significant role in the production of mature mRNA molecules [175, 176]. YTHDC1 has been shown to regulate mRNA splicing by selectively recruiting or repressing various pre‐mRNA splicing factors [177]. These findings highlight the importance of epigenetic modifications in the regulation of gene expression and the complex interplay between different molecular components involved in pre‐mRNA splicing.

Upon the knockdown of the YTHDC1 gene, it was observed that the residence time of m^6^A mRNA in the nucleus was prolonged. This resulted in an accumulation of transcripts in the nucleus and a depletion in the cytoplasm, indicating that YTHDC1 facilitates the export of m^6^A‐modified mRNA from the cytoplasm to the nucleus [178].

Besides YTH family proteins, the IGF2BP family was also found to have a role in m^6^A modulation. This family comprises three proteins: IGF2BP1, IGF2BP2, and IGF2BP3 [179, 180]. Studies observed that these proteins are capable of binding mRNA molecules that possess an “m^6^A” tag through their KH domain [181]. This binding facilitates the promotion of mRNA translation, safeguarding it from degradation and enhancing its stability [182].

In a word, m^6^A‐modulated gene expression and translation are strictly controlled by a series of proteins under physiological conditions. On the other hand, if there is a problem with m^6^A or its related regulatory proteins, relevant diseases may occur (Figure 3).

## 5 The Role of m^6^A in Metabolic Diseases

The interplay between human genetics, nutrient excesses, and lifestyle can lead to metabolic disturbances that increase the risk of developing life‐threatening metabolic diseases such as T2D, obesity, and NAFLD [7, 183, 184]. These diseases not only affect the physical health of patients but also increase the risk of complications such as cardiovascular and cerebrovascular diseases [185]. In terms of clinical strategies, it mainly includes lifestyle intervention, drug treatment, and surgical treatment [186]. Lifestyle intervention, such as reasonable diet, moderate exercise, smoking cessation, and limited alcohol consumption, is the basis for the prevention and treatment of metabolic diseases [12, 187]. Drug therapy is always recommended by clinicians once lifestyle intervention is unsatisfactory, insulin or oral hypoglycemic drugs against diabetes [10, 188], and statin against hyperlipidemia [189, 190]. Surgical treatment is mainly aimed at specific diseases such as obesity [191, 192, 193]. However, existing drugs have certain limitations and may induce side effects, such as hypoglycemia, gastrointestinal discomfort, and abnormal liver function [194, 195, 196]. On the other hand, long‐term use of certain drugs may lead to the development of resistance, resulting in decreased efficacy. In this sense, exploring new strategies against diabetic diseases is necessary [197, 198].

As discussed above, m^6^A modification plays a crucial role in regulating mRNA function and is involved in a variety of physiological processes. Its inherent characteristics make it a potential target different from existing targets: (1) The reversibility of m^6^A modification makes it highly flexible in the cell, and this characteristic makes it an important link in RNA metabolism and plays a key role in a variety of biological processes. (2) m^6^A modification has an important impact on gene expression, and it involves both basic life activities of cells and complex phenotypes of the body. (3) It is associated with a variety of diseases, such as cancer, nervous system diseases, and metabolic diseases. (4) Compared with other RNA modifications, research on m^6^A modification makes it easier to conduct in‐depth studies using in vitro or in vivo models at present, which makes it possible to develop chemical inhibitors against m^6^A modification.

In summary, the remarkable reversibility of m^6^A RNA modification, its extensive role in gene expression regulation, its association with various diseases, and its susceptibility to chemical interference distinguish it fundamentally from existing targets in both biological functions and potential applications. As research progresses and technology advances, m^6^A modification is anticipated to emerge as a pivotal target for the development of new generations of drugs and treatments, heralding a new era in the conquest of major human diseases [51, 52, 199].

### 5.1 m^6^A Methylation and Obesity

With the widespread adoption of Western food culture, there is a significant obesity prevalence [200], leading to an increased risk of chronic diseases such as diabetes and cardiovascular conditions [201]. As a result, public health initiatives and lifestyle changes are becoming increasingly necessary to mitigate these adverse effects on society. Obesity manifests not merely as an appearance of a bloated body; it is a significant predisposing factor elevating the risk of various metabolic diseases, including diabetes, hypertension, and cardiovascular conditions, thereby underscoring the critical need for preventive measures. When excess body fat accumulates, a range of physiological functions are affected [202]. Antiobesity drugs often fall short in their effectiveness and safety, making surgery the most effective treatment option for reducing weight, but surgical treatment of obesity also carries some risks [203, 204]. Therefore, understanding the epidemiology of obesity and its metabolic complications is crucial in identifying new treatments and targets for obesity and related metabolic diseases [205]. Previous research has highlighted the regulatory role of m^6^A modifications in obesity‐related biological processes [206]. In this chapter, we will provide an overview of the progress made in understanding m^6^A modifications in obesity, presenting a new perspective for addressing this global public health concern.

Promoting the browning of white adipocytes is an effective strategy to increase energy expenditure and combat obesity. Studies have shown that m^6^A modification influences the browning and thermogenesis of white adipocytes, offering a potential target for counteracting obesity and metabolic diseases [207]. For instance, Chen et al. found that curcumin effectively inhibited adipogenesis by modulating the ubiquitination of ALKBH5‐m6A‐YTHDF1 rearrangement, demonstrating that m^6^A methylation plays a key role in curcumin‐induced adipogenesis suppression. They proposed that curcumin could be used in dietary supplements to prevent obesity [208]. Moreover, the occurrence and development of obesity involve abnormalities in multiple signaling pathways, with close interaction between m^6^A modification and these pathways [209]. For example, m^6^A modification can affect the activity of the AMPK signaling pathway, a key pathway that regulates cellular energy metabolism and plays an important role in obesity and metabolic diseases [210]. Additionally, m^6^A modification is related to the insulin signaling pathway, jointly regulating the occurrence and development of obesity [211, 212].

The first m^6^A demethylase in eukaryotic cells was identified as FTO. Demethylation of FTO is essential for lipid formation [213, 214, 215]. Research has shown that simultaneous knockout of the m^6^A demethylase FTO reduces ATG5 and ATG7 expression, leading to decreased autophagy vesicle formation and inhibiting adipogenesis [216]. Wang et al. [217] found that NADP can significantly bind to FTO, enhancing its activity and promoting m^6^A demethylation and adipogenesis. FTO promotes adipogenesis by regulating the adipogenic pathway and inducing preadipocyte differentiation. Knockdown of FTO resulted in a decrease in adiposity and body weight in mice, demonstrating that FTO plays a crucial role in promoting adipogenesis by regulating the adipogenic pathway and inhibiting preadipocyte differentiation [218]. In addition, m^6^A‐YTHDF2‐FTO may play a crucial role in the development of obesity. When FTO is silenced, YTHDF2 binds to Atg5 and Atg7 transcripts, leading to reduced mRNA degradation and protein expression. This results in decreased autophagy and adipogenesis, which contribute to obesity [216]. These findings suggest that targeting m^6^A‐YTHDF2‐FTO may be a potential therapeutic strategy for obesity.

Qin and colleagues further demonstrated that deleting METTL3 prevented inflammatory and metabolic phenotypes and ameliorated obesity in mice [219]. Overall, these findings highlight the importance of METTL3 in regulating brown fat development and metabolism and suggest that targeting this gene may be a promising approach for treating obesity and related metabolic disorders.

To summarize, the methylase of m^6^A plays a crucial role in adipogenesis, and m^6^A modification could be a promising new biomarker for obesity (Table 2). At present, research on m^6^A modification in the context of obesity is still in a nascent stage. While some important findings have been achieved, many areas remain unexplored [156]. For instance, the precise mechanism by which m^6^A modification influences metabolic diseases associated with obesity is not fully understood. Furthermore, the development of new antiobesity treatments through the regulation of m^6^A modification requires further exploration. Additionally, there is a need for more studies to clarify the interaction between m^6^A modification and other cell signaling pathways, as well as the impact of environmental factors on m^6^A modification.

### 5.2 m^6^A Methylation and T2D

In today's world, diabetes has emerged as a major public health problem, posing a serious threat to human health. This chronic condition affects millions globally, leading to a myriad of complications such as cardiovascular diseases, kidney failure, and neuropathy. The rise in obesity rates, coupled with sedentary lifestyles and poor dietary habits, has exacerbated the prevalence of diabetes. Early detection and management are crucial for mitigating its impact, yet access to healthcare and education remains a significant challenge for many affected individuals. Addressing this epidemic requires comprehensive strategies involving medical interventions, lifestyle modifications, and community awareness programs to foster healthier living habits. T2D, the most common form of diabetes, accounts for about 90% of all diabetes cases [220, 221, 222]. Its incidence is steadily increasing, posing a global health concern. T2D not only requires long‐term medication or insulin injection to control blood glucose but also causes a series of serious complications, such as diabetic nephropathy, diabetic retinopathy, diabetic liver disease, and other complications. [223, 224, 225]. The pathogenesis of T2D is complex and closely related to many factors. Most cases of T2D are secondary to obesity and its associated insulin resistance (IR) [7, 226]. Pancreatic beta cells have long been considered the main regulators of blood glucose. However, when insulin secretion fails to compensate for IR in peripheral tissues, T2D develops [227, 228]. Currently, metformin is the primary drug used to treat T2D [229, 230]. Newly‐developed drugs including DPP‐IV and SGLT‐2 inhibitors have witnessed effect on reducing blood glucose. However, long‐term application of these drugs induced efficacy reduction and side effect increment such as diarrhea and urinary tract infection. Therefore, it is still necessary to explore new anti‐type 2 diabetes methods and drugs.

Recent studies suggested a potential role of m^6^A in the development of T2D [231, 232, 233]. In hepatocytes, alterations in m^6^A modification can influence the expression of genes involved in critical metabolic pathways, such as glycogen synthesis and gluconeogenesis, thereby exacerbating insulin resistance [234, 235]. Studies on insulin‐resistant animal and cell models have demonstrated that modulating the activity of enzymes associated with m^6^A modification can ameliorate insulin resistance, offering a potential therapeutic target for diabetes treatment related to insulin resistance [212, 236, 237]. For instance, Jiao et al. [238] showed that quercetin enhanced glucose uptake, mitigated oxidative stress, and improved insulin resistance through METTL3 regulation. Additionally, the onset and progression of diabetic complications are influenced by various factors, including m^6^A methylation [239, 240, 241]. Li and colleagues found that m^6^A modification plays a crucial role in high glucose‐induced glomerular endothelial cell damage and diabetic nephropathy [242]. Furthermore, Meng et al. [243] identified a novel function of m^6^A methylation and long noncoding RNA (lncRNA) regulation in pyroptosis and diabetic cardiomyopathy.

Similar findings are reported in human individuals. Yang et al. [244] observed that individuals with T2D exhibit decreased m^6^A levels and heightened expression of FTO, METTL3, METTL14, and WTAP mRNA. Additionally, they found a positive correlation between FTO and glucose levels. This finding was further validated in HepG_2_ cells, where high glucose concentrations led to an increase in FTO protein levels. This was further validated in patients with T2D that the level of m^6^A methylated RNA and METTL3 was consistently elevated in liver tissue [245], a finding that was replicated in mouse experiments. Silencing METTL3 led to a reduction in fatty acid synthase m^6^A methylation and total mRNA levels, thereby suppressing fatty acid metabolism. A recent study [246] reported that METTL3 is also increased in podocytes from patients with diabetic nephropathy, as observed in renal biopsy samples. While knockdown of METTL3 significantly reduced podocytes’ injury and proteinuria in diabetic mice, a finding was further confirmed in high glucose HG‐stimulated podocytes.

Besides METTL3, METTL14 expression was also found to be upregulated in glomerular endothelial cells cultured with kidney tissue from patients with diabetic nephropathy combined with high glucose levels. Furthermore, overexpression of METTL14 increased apoptosis and the inflammatory response of glomerular endothelial cells, leading to worsened kidney injury in DN mice [247].

Furthermore, the m^6^A reader IGF2BP2 has been found to enhance the expression of PDX1, thereby promoting pancreatic β‐cell proliferation and insulin secretion [248]. Converging findings suggest that m^6^A RNA modifications play a role in the regulation of T2D via a mechanism different from existing drugs (Table 3). Currently, although proteins associated with m6A modification are anticipated to serve as potential targets for diabetes treatment, there are several limitations in the mechanisms of action research, study models, clinical application translation, and technical methodologies. Future efforts require further in‐depth investigation to overcome these constraints, thereby facilitating the advancement and practical application of m6A modification research in the context of diabetes.

**TABLE 3:** The role of m^6^A modification in T2D.

m6A methylase	Main functions	References
FTO	Upregulation of blood glucose may be associated with the expression of related genes that promote lipid and glucose metabolism.	[244]
METTL3	Promotes fatty acid metabolism by elevating m^6^A methylation and total mRNA levels of fatty acid synthase. Promotes foot cell injury and proteinuria in STZ‐induced diabetic mice.	[245] [246]
METTL14	Promotes glomerular endothelial cell apoptosis and inflammatory response in DN mice, and aggravates renal injury.	[247]

### 5.3 m^6^A Methylation and NAFLD

NAFLD has become a high‐profile public health problem worldwide, the incidence of NAFLD is about 25% [249, 250]. Numerous studies have confirmed each other, fully showing the widespread existence of NAFLD in the population. It has been recognized as one of the main causes of chronic liver disease, and this conclusion has been strongly confirmed in many authoritative research literature; the burgeoning incidence of NAFLD underscores the urgent need for public health interventions to mitigate its impact on global health [251, 252]. NAFLD has unique pathological features, and its significant hallmark is steatosis, that is, excessive fat accumulation in liver cells, while the patient does not have a history of excessive alcohol consumption, which has been clearly defined in the literature [253, 254].

The disease is not static, and if it is not effectively treated and intervened in the early stages of the disease, it will gradually develop in a more severe direction. After the disease progresses, the patient may develop steatohepatitis, the inflammatory response of the liver is aggravated, and the liver cells are further damaged. As the course of the disease continues, cirrhosis may also follow. Liver tissue gradually becomes fibrotic, normal liver structure is destroyed, and liver function is severely impaired. Most seriously, some patients will even deteriorate into hepatocellular carcinoma, which directly threatens the life and health of patients [255, 256, 257].

Recent research conducted on NAFLD mice through immunoprecipitation sequencing and RNA transcriptome sequencing of methylated RNA has shown that m^6^A modification is positively associated with this disease [258]. This suggests that m^6^A methylation modification may play a crucial role in the development and progression of NAFLD. For instance, Jiang *et al.* have shown that Arbutin can ameliorate high‐fat diet (HFD)‐induced NAFLD via the FTO/SLC7A11 pathway; the mechanism of action involves inhibition of Fatty Acid Transcriptional Repressor (FTO), which in turn enhances m^6^A methylation of Solute Carrier Family 7 Member 11 (SLC7A11); this process upregulates SLC7A11 expression and ultimately inhibits ferroptosis, offering a novel approach and theoretical foundation for NAFLD treatment [259, 260, 261, 262].

Regarding liver inflammation, m^6^A RNA methylation is significantly associated with glucose and lipid metabolism disorders and the exacerbation of liver inflammation [263]. T2D is closely related to NAFLD through its effects on lipid metabolism, potentially leading to NASH, fibrosis, and hepatocellular carcinoma (HCC). It has been demonstrated that m^6^A RNA methylation is an important epigenetic regulator of gene expression and is related to the occurrence of HCC [15]. Additionally, research has shown that elevated m^6^A modification effectively inhibits hepatic lipid accumulation, while inhibition of m^6^A modification leads to hepatic lipid deposition. These findings highlight the beneficial role of m^6^A RNA methylation in hepatic lipid metabolism, which may protect the liver from lipid metabolic disorders [264]. Further study found that dysregulation of m^6^A methylation contributes to steatosis and fibrosis, thereby affecting the development of NAFLD [265].

Previous studies [266] have demonstrated that the demethylation function of FTO plays a crucial role in hepatocyte lipid metabolism. In mice fed a high‐fat diet, decreased liver m6A RNA methylation was observed alongside increased expression of the FTO gene; furthermore, overexpression of FTO in the liver led to enhanced triglyceride accumulation by upregulating the expression of lipogenic genes [267]. The association between epigenetic modifications of RNA and fat deposition has provided a new target for regulating hepatic lipid metabolism. FTO is overexpressed in the liver, which promotes adipogenesis and lipid droplet expansion. Additionally, cpt1‐modified fatty acid oxidation is inhibited, leading to excessive lipid storage and the development of NAFLD [268]. Researchers have used animal models to show that downregulation of FTO significantly alleviates dexamethasone‐induced fatty liver in mice. The effect of FTO's reverse transcriptional activation and m^6^A demethylation in NAFLD pathogenesis has been demonstrated [269].

A recent study published in 2022 unveiled a pathway by which m^6^A methylation affects hepatocyte lipid metabolism through the regulation of autophagy [270]. By constructing both in vivo and in vitro models of NAFLD, researchers observed that m^6^A modification as well as METTL3 and YTHDF1 expression were significantly elevated in steatosis hepatocytes. Furthermore, they found that hepatocyte autophagic activity was significantly reduced in these cells. Interestingly, when METTL3 was knocked down, it led to an increase in lipid droplet clearance in the liver. This study sheds light on the intricate relationship between m^6^A methylation and hepatocyte lipid metabolism and highlights the potential therapeutic benefits of targeting this pathway in the treatment of NAFLD.

YTHDC2 plays a crucial role in regulating hepatic lipogenesis [271], as evidenced by its significant downregulation in the livers of NAFLD patients and obese mice. This observation was further validated through experiments demonstrating that YTHDC2 overexpression can alleviate hepatic steatosis and insulin resistance. These findings on m^6^A methylation in NAFLD offer promising insights into understanding the progression of this disease (Table 4). In summary, the m^6^A modification plays a crucial regulatory role in the onset and progression of NAFLD, encompassing multiple pivotal processes such as liver fat metabolism and inflammatory responses [234, 259, 272]. However, current research on m^6^A modifications in the liver remains limited. As investigations progress, it is anticipated that they will elucidate the function of m^6^A modification in the pathogenesis of fatty liver and offer new therapeutic possibilities for patients with this condition.

**TABLE 4:** The role of m^6^A modification in NAFLD.

m6A methylase	Main functions	Reference
FTO	Promotes excessive lipid storage and NAFLD via inhibition of CPT‐1‐modified fatty acid oxidation. Promotes fatty liver in mice, probably via GR‐mediated activation of FTO reverse transcription.	[268] [269]
METTL3	Promotes the formation of NAFLD via the inhibition of hepatic autophagic flux and lipid droplet clearance.	[270]
YTHDC2	Inhibits hepatic steatosis and insulin resistance in obese mice.	[271]

### 5.4 Potential Application of M^6^A Modification in Metabolic Diseases

As the prevalence of metabolic disorders continues to escalate globally, identifying effective therapeutic targets and biomarkers has emerged as a focal point in research [273]. Investigating the potential utility of m^6^A modifications in conditions such as diabetes, obesity, and nonalcoholic fatty liver disease is of paramount importance. This includes exploring its use not only as a novel therapeutic target for drug development but also as a diagnostic, monitoring, and prognostic biomarker [274].

Significant strides have been made in exploring m^6^A modification as a therapeutic target. In the context of diabetes mellitus, inhibiting METTL3 activity has been shown to diminish the m^6^A modification levels of genes associated with insulin resistance, consequently enhancing insulin sensitivity [275]. Similarly, in NAFLD, inhibition of METTL3 has demonstrated potential in mitigating liver steatosis [145]. Currently, the pursuit of small molecule inhibitors targeting METTL3 has garnered substantial attention [276, 277]. While certain compounds have exhibited promising therapeutic effects in both cellular and animal models, further refinement and clinical trials are imperative for their validation.

The m6A modification also holds potential for the diagnostics of metabolic disorders [278]. In diabetic populations, alterations in the m^6^A modification levels of certain metabolism‐related gene mRNAs in blood have been observed [279, 280]. Specifically, variations in the m^6^A modification levels of genes implicated in insulin secretion and action could serve as promising biomarkers for the early detection of diabetes. Additionally, the m^6^A modification levels of several adipokine genes in serum correlate with obesity severity, offering a useful reference index for obesity diagnosis [281, 282]. Furthermore, the expression levels of m^6^A modification‐associated proteins, such as METTL3 and FTO, in the blood may also be indicative of metabolic disease onset, thereby providing auxiliary diagnostic value [283, 284].

In NAFLD, the overall m^6^A modification level in liver tissue and the specific gene modification patterns are closely associated with disease severity [263]. The assessment of m^6^A‐related indicators in liver biopsy samples, including m^6^A modification levels and the expression of methyltransferases and demethylases, facilitates precise diagnosis of NAFLD and differentiation among its stages, such as simple fatty liver and nonalcoholic steatohepatitis. During diabetes progression, continuous monitoring of blood or tissue‐specific m^6^A modification dynamics allows real‐time tracking of disease advancement [285]. For instance, as diabetic nephropathy advances, there is a gradual increase in the m^6^A modification levels of fibrosis‐associated genes in kidney tissue. Timely detection of these indicators enables a timely understanding of renal lesion severity [286]. Monitoring the expression alterations of m^6^A modification‐related genes in adipose tissue enables the assessment of obesity‐related complications, including cardiovascular disease risk. Additionally, m^6^A modification‐associated markers are instrumental in evaluating the prognosis of patients with metabolic disorders. In the context of NAFLD, elevated expression of METTL3 in liver tissues correlates with an increased likelihood of disease progression to cirrhosis and hepatocellular carcinoma [15, 141, 287]. Consequently, METTL3 expression levels serve as a crucial prognostic indicator for NAFLD patients. For diabetic individuals, specific gene m^6^A modification profiles are linked to both therapeutic responsiveness and the incidence of complications [85, 288], thereby aiding in the prediction of patient outcomes and facilitating the development of tailored treatment strategies.

In summary, m^6^A modification holds significant potential for applications in metabolic diseases [289, 290]. It offers novel perspectives and directions for the prevention and treatment of these disorders, whether utilized as a therapeutic target for drug development or as a biomarker for diagnosis, disease monitoring, and prognosis assessment [291]. Nonetheless, research in this area remains nascent, presenting numerous challenges and issues. Regarding therapeutic targets, while theoretically feasible to intervene with m^6^A modification‐related proteins, practical applications necessitate addressing issues of drug specificity, efficacy, and safety [237]. For instance, small molecule inhibitors or activators may exhibit off‐target effects, impacting other normal physiological functions. Furthermore, the function of m^6^A modification varies across different tissues and cell types, highlighting the urgency to achieve precise targeted therapy. In the context of biomarkers, although some m^6^A modification‐associated indicators have been identified as related to metabolic diseases, their reliability, and accuracy require further validation through large‐scale clinical studies [289, 290]. Concurrently, establishing standardized detection methods for widespread clinical use is imperative.

## 6 Conclusion and Perspectives

Over the past few decades, RNA modifications in cancer have been extensively studied which further resulted in significant advancements in epigenetics [36, 161, 292, 293], but its role in metabolic diseases is still largely unknown. RNA modification encompasses various forms, and in metabolic diseases, aberrant RNA modifications can influence gene expression [294, 295]. m^6^A is a dynamic and reversible modification that plays a crucial role in regulating gene expression, RNA splicing, RNA stability, and RNA editing [101, 296]. Since the identification of FTO [112], several studies have shown the involvement of m^6^A modifications in the pathogenesis of metabolic diseases such as T2D, obesity, and NAFLD [1, 297]. The development of m^6^A detection technology further facilitates the discovery of its involvement in these diseases [107, 298, 299]. This review delves into the pivotal role of RNA modifications in metabolic pathways, encompassing a diverse array of RNA modification types, with a particular emphasis on m^6^A modifications. To facilitate comprehension, the paper commences by outlining the various forms of modified RNA and subsequently summarizing non‐M^6^A modifications associated with metabolic disorders. It then proceeds to provide an in‐depth exploration of the discovery history of m^6^A, highlighting both the merits and drawbacks of contemporary prevalent m^6^A detection techniques. Furthermore, it reviews the biological processes that are regulated through RNA modification.

The discovery and advancement of m^6^A methylation have spanned several decades [300, 301, 302]. This modification is carried out by a group of enzymes that work in tandem to alter RNA expression, which subsequently impacts the function of cells and ultimately the entire organism [303, 304, 305]. Over time, novel m^6^A assay methods have been developed to facilitate the detection and quantification of this modification. However, each method has its inherent advantages and limitations. In 2023, GLORI, a newly developed m^6^A detection technology, appears and provides the ability to identify m^6^A at the single‐base level. However, the study of m^6^A in metabolic diseases is still relatively limited compared with its understanding in the field of oncology [48, 306]. Further exploration is required to understand how m^6^A methyltransferase and demethylase maintain a dynamic balance in healthy organisms, as well as to uncover the specific molecular mechanisms underlying m^6^A modification in metabolic diseases [17, 52, 273].

In the field of life science research, exciting news is constantly emerging. In recent years, m6A modification has gradually come into people's vision and is considered to have shown great potential in the treatment of some diseases [148]. These diseases span a wide spectrum, including certain types of cancer, neurodegenerative diseases, and metabolic diseases [4, 45, 278, 307]. As one of the most common forms of RNA modification, m6A modification plays a key role in the regulation of gene expression. In the field of cancer research, a large number of studies have shown that the abnormal expression of m^6^A modification‐related proteins is closely related to the occurrence and development of tumors. For example, in some tumors, high expression of m^6^A methyltransferases promotes tumor cell proliferation, invasion, and metastasis, whereas inhibition of the activity of these enzymes has the potential to inhibit tumor growth. This finding provides new targets and ideas for cancer treatment.

In neurodegenerative diseases, m^6^A modification is also involved in the pathological process of diseases. For example, Alzheimer's disease, Parkinson's disease, and so on; related studies have found that abnormal changes in m^6^A modification can affect the expression of related genes in nerve cells, leading to the dysfunction and death of nerve cells. Therefore, improving the function of nerve cells by regulating m^6^A modification has become a potential strategy for the treatment of neurodegenerative diseases. In the field of metabolic diseases, with the rising incidence of obesity, diabetes, and other metabolic diseases worldwide, it is urgent to find effective treatment methods. The research of m^6^A modification in metabolic diseases has gradually deepened, showing its potential in treatment. For example, the role of m^6^A modification has become increasingly prominent in the study of diabetes and its related complications.

Currently, a handful of visionary pharmaceutical development entities and leading research institutions have astutely identified the potential of m^6^A modification in addressing metabolic diseases, actively pursuing drug development initiatives anchored in this molecular mechanism to pioneer novel therapeutic strategies for metabolic disorders [293, 308, 309]. However, it is crucial to maintain a realistic perspective acknowledging that these efforts are still nascent. While theoretical advancements and laboratory breakthroughs have been realized, the path to their clinical application remains protracted. Substantial further research, coupled with rigorous clinical trials, is imperative to definitively establish both the efficacy and safety profiles of these promising agents. Recently, METTL3 has been found to be elevated in kidney biopsies of patients with diabetic nephropathy and has been verified in mice. Knockdown of METTL3 significantly reduced podocytes’ damage and proteinuria in STZ‐induced diabetic mice [310]. Therefore, it is reasonable to hypothesize that the deployment of METTL3 inhibitors could serve as a therapeutic avenue to diminish METTL3 levels and mitigate diabetic nephropathy to a certain degree. METTL3 stands out as a pivotal enzyme instrumental in the m^6^A methylation pathway [311, 312]. While indispensable for the onset and progression of diseases, its fundamental role in preserving cellular homeostasis cannot be overstated [313, 314]. Targeting m^6^A modification or directly suppressing METTL3 activity may offer therapeutic advantages, yet such interventions could potentially disrupt the equilibrium of intricate cellular processes. This disruption might provoke a cascade of adverse events, jeopardizing not only cellular health but also the overall well‐being of the organism. Therefore, it is particularly important to eliminate the off‐target effects of m^6^A modification‐related drugs when developing therapeutic drugs based on m^6^A modification or targeting METTL3. Only in this way, we can effectively treat the disease while minimizing the adverse effects on the normal physiological function of the body, and bring a truly safe and effective treatment plan for patients.

In summary, the pivotal role of epigenetic modulation in disease progression has been firmly established over the past decade. RNA methylation, as a predominant form of epigenetic regulation, has garnered substantial interest and has undergone extensive investigation in recent years [315, 316]. Within this spectrum of RNA methylation modifications, m^6^A modification emerges as a particularly notable entity. An abundance of research underscores the indispensable function of m^6^A in modulating biological processes and its intimate association with the onset of diverse diseases [48, 317, 318]. Of particular note is the remarkable advancement in detection technologies for m^6^A modifications in recent times. Techniques such as m^6^A‐seq and miCLIP have enabled more precise identification and localization of m^6^A sites on RNA molecules, facilitating in‐depth analysis of alterations in modification levels [140, 319, 320]. These advancements not only enhance our understanding of the functional mechanisms of m^6^A under physiological conditions but also illuminate its critical involvement in disease development, thereby furnishing a potent toolkit for elucidating disease pathogenesis. As the global population ages at an accelerated rate and Western dietary habits proliferate globally, there is a stark rise in metabolic disease incidence worldwide [321, 322]. Diseases such as diabetes mellitus, obesity, and NAFLD pose significant health risks and impose substantial societal and familial burdens. Consequently, there is immense practical importance in actively seeking novel prevention and therapeutic strategies for these metabolic conditions [323, 324, 325]. Advancements in RNA modification research have increasingly highlighted RNA modifications, particularly m^6^A methylation, as promising therapeutic targets for metabolic disorders [14]. Efforts are underway to develop targeted medications for proteins involved in m^6^A metabolism, including methyltransferases, demethylases, and readers. These drugs hold promise for correcting aberrant gene expression in metabolic diseases by modulating m^6^A levels, thereby offering avenues for effective treatment [297, 326]. While RNA modifications have demonstrated substantial promise in treating metabolic diseases, it is imperative to maintain a nuanced understanding of their potential impacts. Given that RNA modifications are intricately involved in numerous biological processes within organisms, targeted interventions may inadvertently disrupt normal physiological equilibrium, potentially leading to unforeseen side effects. Consequently, the advancement and clinical implementation of RNA modification‐based therapies remain fraught with challenges [327]. To navigate these complexities, it is crucial to deepen the comprehension of RNA modification mechanisms and refine drug development strategies, prioritizing both the safety and efficacy of therapeutic interventions.

## Author Contributions

Yadi Liu and Zhongyan Sun wrote the initial draft. Dingkun Gui and Yonghua Zhao helped constructive discussions. Youhua Xu reviewed the manuscript and provided suggestions for revision. All the authors have approved the final manuscript.

## Conflicts of Interest

The authors declare no conflicts of interest.

## ETHICS APPROVAL

Not applicable.

## Data Availability

The authors have nothing to report.
